# Relationship between the strength of the ankle and toe muscles and functional stability in young, healthy adults

**DOI:** 10.1038/s41598-024-59906-7

**Published:** 2024-04-21

**Authors:** Kajetan J. Słomka, Justyna Michalska

**Affiliations:** Institute of Sport Sciences, Academy of Physical Education in Katowice, Mikolowska 72A, 40-065 Katowice, Poland

**Keywords:** Limits of stability, Force plate, Maximal isometric force, Center of foot pressure, Muscle strength, Motor control, Musculoskeletal system

## Abstract

This study investigates the relationship between ankle and toe strength and functional stability in young adults, with a sample comprising sixteen females and fourteen males. The research employed force platform data to determine the center of foot pressure (COP) and calculated the forward functional stability index (FFSI) through foot anthropometric measurements. Strength measurements of toe and ankle muscles, during maximal isometric flexion and extension, were conducted using force transducers. Notable positive correlations were found between toe flexor strength and FFSI (left flexor: r = 0.4, right flexor: r = 0.38, *p* < 0.05), not influenced by foot anthropometry. Contrarily, no significant correlation was observed between ankle muscle strength and FFSI, despite a positive correlation with the COP range. The moderate correlation coefficients suggest that while toe flexor strength is a contributing factor to functional stability, it does not solely determine functional stability. These findings highlight the critical role of muscle strength in maintaining functional stability, particularly during forward movements and emphasize the utility of FFSI alongside traditional COP measures in balance assessment. It is recommended to employ a multifaceted approach is required in balance training programs.

## Introduction

Multiple elements contribute to the body’s equilibrium. The relative importance of these factors for maintaining an upright posture varies, with the vestibular system and proprioception accorded the greatest significance. The responsiveness of these systems is responsible for the body making rapid adjustments and responding to environmental disturbances^1^. The final component of the movement system involves muscles that carry out programmed automatic reactions or voluntary movements^2^. Their efficacy is responsible for the proper execution of the processes maintaining the body’s equilibrium. Postural muscles—including triceps surae (calf muscles), gluteus maximus, major lumbar, erector spinae, suboccipitals, obliquus capitis (oblique neck muscles), abdominal oblique and rectus muscles, quadriceps femoris, and tibialis anterior—are involved in almost every global body movement. This encompasses the synergistic action of multiple muscle groups and joints to perform complex motions, to a greater or lesser extent, and is the first element involved in maintaining the body's posture^3^. The body’s equilibrium can be evaluated through clinical and laboratory studies. These are, in most cases, an objective source of knowledge about the state and any stability-related shortcomings. In laboratories, computerized posturography is most frequently employed for both static and dynamic laboratory examinations of balance^4^. In most studies, the term static balance is used for quiet standing trials while the term dynamic balance is used when a person is performing a specific task, e.g., leaning in different directions. However, according to the biomechanical definition, a person remains in a static state as long as the support plane does not change. Therefore, to differentiate between these two static condition tasks, it is advisable to employ the term “functional balance” instead of “dynamic balance”. An example of a functional balance test is the maximum leaning test or limits of stability (LOS) test. Leaning in various directions relative to the target or at the participant's discretion within their maximum capabilities are variations of this test^5^. These tests can also be more specific, such as leaning in a particular way, to establish the limit of stability in that direction. Such a test is the forward limits of stability test^6,7^. Previous studies have shown that the results of LOS tests provide more clarity regarding falls and fear of falling in older adults, in people with Parkinson's disease (PD)^8,9^. A few studies^10,11^ have also found that the results of a static equilibrium test and a maximum deflection test do not agree very well. This disagreement motivates research toward more functional measurements and also paves the way for more specific measurements of muscle strength. Falls in older adults—often attributed to progressive sarcopenia, a condition characterized by the loss of muscle mass, strength, and function^12,13^—represent a significant health concern. Consequently, there is an imperative need for more precise tests and comprehensive intervention programs that can substantially improve quality of life, enhance mobility and balance, and reduce the risk of falls.

In the context of the correct implementation of the LOS test, the efficiency of each of the vestibular, proprioception, and muscle systems is substantial. An important of this test is assessing whether the musculoskeletal system of the ankle joint complex and the muscles of the toes work effectively at the same time. The muscles in the toes supply the necessary force for maintaining anterior–posterior stability, especially while leaning forward, and contribute to the proprioceptive feedback loop, which is essential for balance. Proprioception is the sense of the relative position of one's body parts and the amount of effort expended in movement. Sensory receptors in the toe muscles and joints offer crucial information to the central nervous system about the position and motion of the body, enabling the adjustments needed to maintain balance. They provide a counterforce to the forward lean, assisting in the prevention of a forward fall^14,15^. While the intricate muscles of the ankle joint, including those in the calf, foot, and toes, may not always be in the spotlight, their role remains crucial. Beyond mere anatomy, one must underscore the profound impact these muscles have on our daily lives—enabling us to walk, run, balance, and perform essential tasks. Recognizing their significance ensures a deeper understanding of our body’s intricate mechanics.

Insufficient muscle strength in the ankle joint and the toes of the foot makes it impossible for a person to perform fast postural movements like forward reaching and restricts their functional ability to reach as far as possible^16,17^. If a correlation is found between the strength of the muscles of the ankle complex and the muscles of the toes, it would enable the development of systemic remedies to avoid falls through the targeted strengthening of the relevant muscle groups.

Previous research on the effects of toe muscles and the ankle joint on balance suggests that strong connections exist between these factors. Tanaka et al.^14^ demonstrated a significant linear relationship between the parameters of body sway and great toe pressure. Moreover, older adults have been found to use higher toe pressure levels more frequently than the younger group, presumably due to an altered posture and a forward shift in older adults’ center of mass (COM)^18,19^. This mechanism contributes to age-related changes in body posture^20^, leading to a consequent deterioration of the equilibrium. Although older people use greater levels of toe pressure, their toe muscles are predictably weaker than those of younger people^17,21^. This can result in a loss of balance and subsequent falls. The effect of toe and ankle strength on functional balance has not been extensively explored or emphasized in the literature. Endo et al.^17^ found notable correlations between the strength of the toe plantar flexors and the anterior limit of the functional base of support. The authors normalized toe flexor strength derived from a force plate during one-legged toe standing to body measurements. There was no direct measurement of toe muscle strength. Menz et al.^22^ used a simple paper grip test to explore the strength of the plantar flexor muscles in the hallux and lesser toes and how they affect balance. The functional balance tests were performed using a sway meter, a simple analogue method that depicts postural sway^23^. Menz et al.^22^ reported findings similar to those of Endo et al.^17^, that muscle function might be crucial in maintaining balance among older individuals. This was further substantiated by the same group in a subsequent study, which also included a range of other important balance indicators^24^.

Chou et al.^15^ also examined the role of the great toe in balancing performance. Their approach included disabling toe function by using a special toe-tying harness in a 30° dorsal bend. The authors found that directional control scores (%) were markedly lower during rhythmic weight shifting between the two toe conditions in the forward/backward direction, whereas this was not the case in the left/right direction.

Though the aforementioned studies showed that toe flexors and lesser toe muscles play a substantial role in functional balance when a person is standing, the studies investigating a similar relationship involving both ankle and toe dorsiflexors are limited in number. Additionally, these studies did not employ direct measurement of toe and ankle strength. These measures were expressed as the pressure exerted on a force plate or normalized to the body height during one-legged standing on a platform. Objective and reliable measurements are essential to confirm these interdependencies. Further, isolated measurement of the toe and ankle muscles can provide a rich understanding of an individual’s balance and stability, particularly in the context of executing tasks that demand a high degree of coordination and equilibrium, such as the LOS test. Though research exploring the impact of the toes and ankle dorsiflexors on functional balance is scarce, their expected role in motor control for maintaining standing posture is noteworthy. Therefore, this study aimed to ascertain how toe and ankle plantar flexors and dorsiflexors affect functional balance. It was hypothesized that the strength of the plantar flexors and dorsiflexors in the toes and ankles is strongly linked to functional balance, which suggests that increased strength correlates with an enhanced range of maximal forward lean. Further, employing both holistic and targeted approaches to measure dorsiflexors and plantar flexors individually, as well as isolated assessments of the ankle and toe separately, would contribute to a deeper understanding of this phenomenon. Researchers and clinicians employ the LOS test as a primary method to assess an individual's balance; the test is deemed essential for evaluating functional capability and its significant correlation with fall risk^25^.

## Methods

### Subjects

G*Power was used to perform a sample size calculation based on the data from the pilot study. The results suggested that a sample size of 29 was sufficient to reach a power of 0.8. Consequently, thirty healthy young individuals (16 women and 14 men) volunteered for the study. Table 1 displays their average age, weight, and height, as well as their anthropometric foot measurements. The general inclusion criteria were age over 18 and no experience in competitive sports or activity involving balance training. The exclusion criteria encompassed no neurological issues influencing balance or musculoskeletal injury with the required surgical intervention received within six months or a foot deformity, e.g., hallux. Each participant provided informed consent by signing a consent form before participating in the study. The research was conducted in accordance with the Declaration of Helsinki and with the approval of the Ethics Committee of the Academy of Physical Education no. KBBN/1/2021.

**Table 1:** Participant characteristics.

Variable	Women (n = 16) Mean ± SD	Men (n = 14) Mean ± SD
Age [years]	22.3 ± 1.8	22.3 ± 1.8
Body height [cm]	164.6 ± 6.5	181.7 ± 5,1
Body weight [kg]	63.1 ± 10.6	79.7 ± 10.1
Foot length [cm]	23.2 ± 1.1	26.1 ± 1.5
Big toe length [cm]	6.0 ± 0.5	6.9 ± 0.8
Forefoot length [cm]	11.9 ± 0.9	13.6 ± 1.1

### Procedures

Three measurement techniques were employed in the investigation, viz., anthropometric foot measurements, plantar and dorsiflex toe and ankle muscle force evaluation, and force platform functional stability testing. All measurements were performed barefoot.

**Figure 1:**
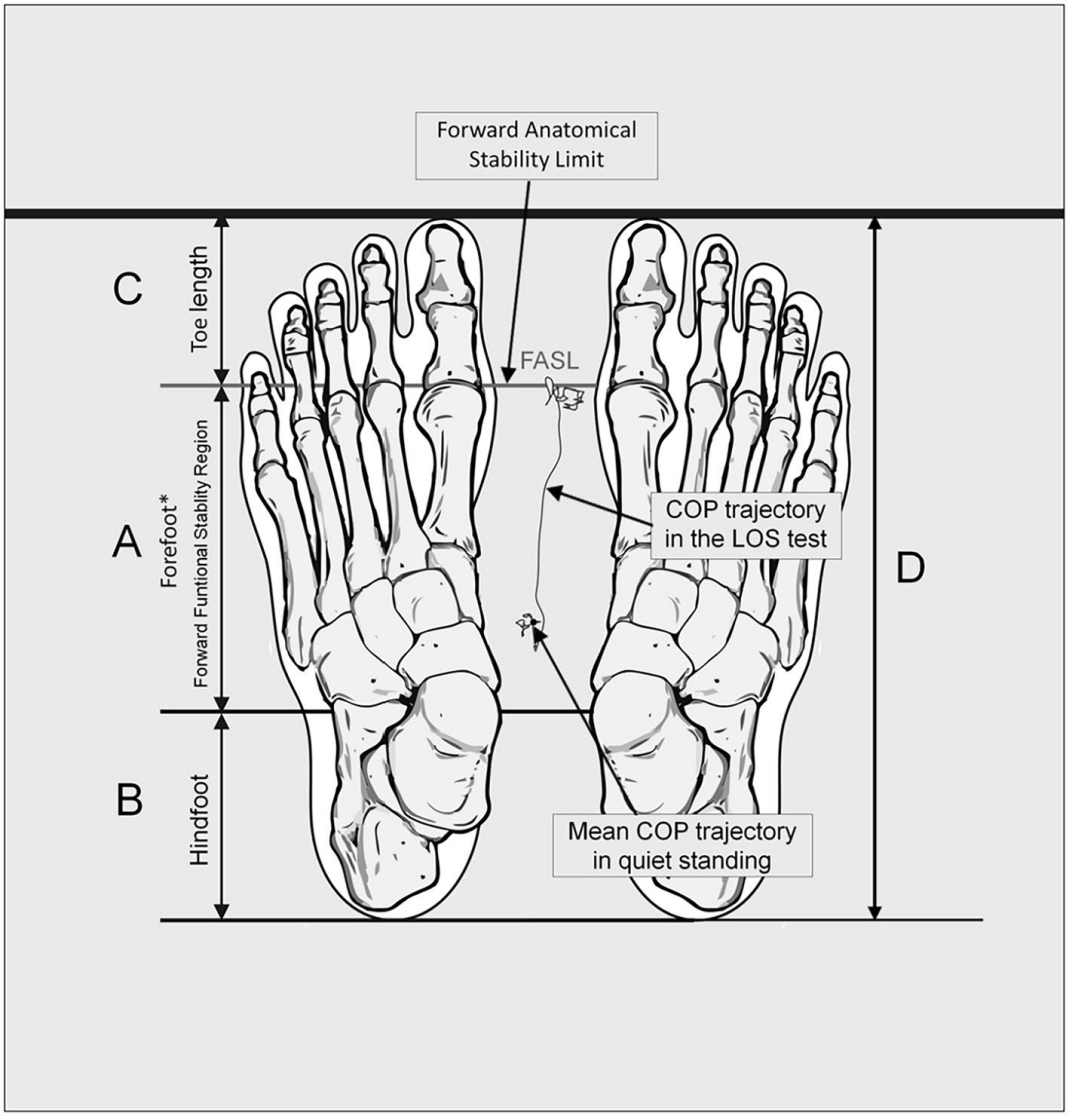
Anthropometric measures of the feet and forward anatomical stability limit (FASL), *forefoot measured from the fifth metatarsophalangeal joints to the middle of the medial malleolus (Słomka et al.^7^).

As detailed by Słomka et al.^7^, initial foot measurements were taken to provide an accurate estimate of the forward functional stability region (Fig. 1). The proposed technique refers to the center of foot pressure (COP) data for individual anthropometric foot measurements. A spreading caliper was used to take the measurements. Two joints in the foot (the top talonavicular joint and the first metatarsophalangeal joint) defined the axis of rotation in the sagittal plane and were used to define the various foot components. The following places on the foot were highlighted with a marker and measured with a spreading caliper: the farthest point on the calcaneus, the center of the medial ankle of the tibia, the first metatarsophalangeal joint, the end of the toe, and the foot's leading point. Based on these measurements, the length of the foot, the length of the toe, and the distance between the medial ankle and the first metatarsophalangeal joint were determined (i.e., the length of the forefoot).

**Figure 2:**
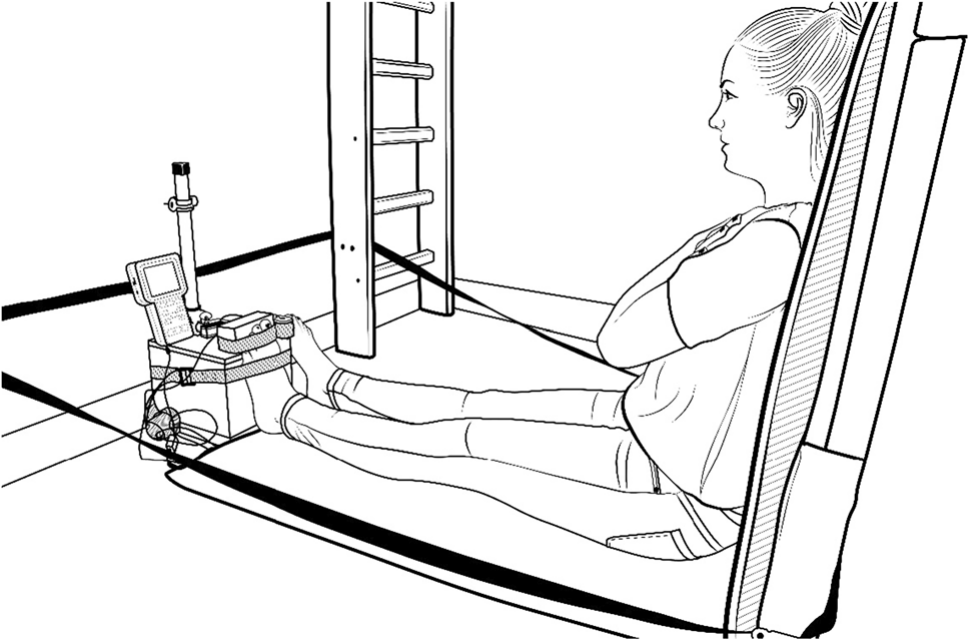
Experimental setup for force measurement and the participant’s seated position with isolated toes on a wooden block.

Next, the maximal isometric flexion and extension forces of toe muscles, as well as ankle joint muscles, were measured. For this, a linear force transducer (SML Low Height S-type load cell, Interface®, Inc. Arizona, USA) with the Myotrace 400 (Noraxon, Inc., USA) measurement system was used. The experimental setup enabled the measurement of the pull and push forces (Fig. 2).

The measurements were conducted in a seated position with straight legs and arms crossed on the chest. This position eliminated the influence of body weight on the flexor force.

**Figure 3:**
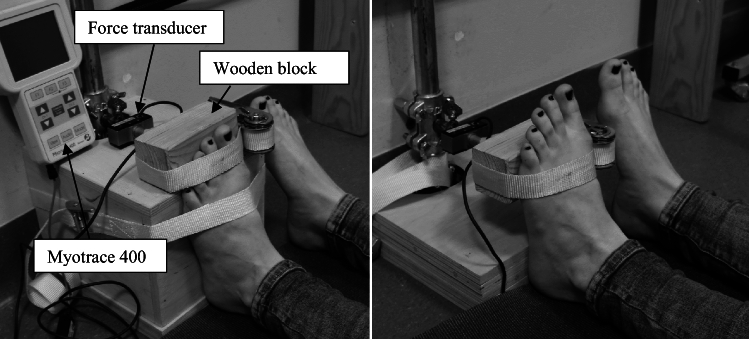
Placement of the foot on the wooden block when measuring toe flexors and extensors (on the left) and ankle flexors and extensors (on the right).

The tested foot rested flat on a wooden base block. The height of the block was adjusted to the line of the first metatarsophalangeal joint so that the toe protruded above the block and was free to move. The linear force transducer was fixed to the wall in front of the participant. Special construction enabled adjusting the height of the force transducer to the size of the subject’s foot. A smaller wooden block was attached to the force transducer on which the toes rested, and force was exerted while testing. Fixation of the foot to the wooden base block at the level of mid-sole with the ratchet tie-down strap was used to stabilize the foot during the test. Similarly, a ratchet tie-down strap was used to fix and stabilize the toe rested on a force transducer wooden block. This enabled the measurement of both the pushing and pulling forces (Fig. 3). The pelvis of each subject was stabilized with another ratchet tie-down strap dragged behind their lower back. The measurement of the ankle joint muscles was done when the wooden base block was removed and the force transducer block was attached to the foot at the level of the midsole.

The testing procedure consisted of four measurement conditions: toe plantar flexion (pushing), toe extension (pulling), ankle joint plantar flexion (pushing), and ankle joint dorsiflexion (pulling). The participants were instructed to exert maximal isometric force in each condition for 5 s. The measurement in each condition was repeated thrice, with a rest break of 2 min to avoid fatigue. A random order of measurement conditions was introduced. The reliability study conducted on the obtained force measures showed excellent reliability (R = 0.9), with only one or two repetitions of the trial (Table 2)^26^.

**Table 2:** Reliability of the force measurements (ICC_2,1_).

Variable	*R* = 0,9		ICC CI	CI	SEM (N)
	Women	Men			
LT_F	1	1	0.95	0.888–0.98	19.18
LT_E	2	2	0.95	0.782–0.957	6.5
RT_F	1	1	0.95	0.9–0.982	17.22
RT_E	2	2	0.95	0.74–0.947	7.15
LA_DF	1	1	0.95	0.935–0.989	9.88
LA_PF	1	1	0.95	0.975–0.996	20.55
RA_DF	1	1	0.95	0.918–0.985	9.91
RA_PF	1	1	0.95	0.87–0.976	44.85

*LT_F* left toes flexion, *LT_E* left toes extension, *RT_F* right toes flexion, *RT_E* right toes extension, *LA_DF* left ankle dorsiflexion, *LA_PF* left ankle plantar flexion, *RA_DF* right ankle dorsiflexion, *RA_PF* right ankle plantar flexion, *ICC* intra class correlation, *CI* confidence intervals, *SEM* standard error of measurement.

Functional balance testing was conducted through a force plate (AMTI AccuGait, Watertown, MA, USA), which registered forces (Fx, Fy, Fz) and moments (Mx, My, Mz) at a 100 Hz sampling frequency. The platform raw data were further processed offline through a 4th-order 7 Hz low-pass Butterworth filter. The processed raw data were used to calculate the COP of the participants.

The LOS test was used to estimate the functional stability limit of the participants^6^. This test—in conjunction with the feet measurements where an individual’s forward anatomical stability limit (FASL) is designated—enables precise estimation of an individual’s functional stability limit. The LOS test consists of three phases: quiet standing, transition to maximum forward-leaning, and maintenance of the inclined position until the end of a trial. In the current study, the participants were standing still on a force platform with their arms at their sides and their eyes focused in front of an object three meters away and positioned at eye level. They were directed to make the maximum leaning forward after the acoustic cue as rapidly as feasible. During the test, the participants were advised to keep their entire foot in continual contact with the platform. The measurement was interrupted and repeated if the participants lifted their heels or changed their body position. Before the evaluation, the participants had the chance to practice the research procedure. The following variables were further analyzed: maximal forward COP displacement (COPrange) and forward functional stability index (FFSI)^7^.

### Statistical analysis

In the initial step of statistical analysis, typical descriptive statistical methods were employed. The Kolmogorov–Smirnov and Lilliefors tests were conducted for the composite hypothesis of normality. Pearson's correlation coefficient r was used to determine the statistical link between anthropometric measures of the feet and ankle force performance, anthropometric feet measures and variables of functional balance, as well as toe and ankle force performance and variables characterizing functional balance. The threshold for statistical significance was established at a *p* value of 0.05. Statistica 13.3 was used for the statistical analysis (TIBCO Software Inc. 2017).

## Results

Several significant correlations related to foot length and its impact on force performance were observed in the study. Specifically, both right and left foot lengths consistently showed significant positive associations with most of the force performance variables, as detailed in Table 3.

**Table 3:** Correlation of toe and ankle force performance with anthropometric variables of the feet (*p < 0.05).

Variable	Foot length		Toe length		Forefoot length	
	Right	Left	Right	Left	Right	Left
LT_F	0.62*	0.56*	0.68*	0.61*	0.34	0.28
LT_E	0.48*	0.51*	0.33	0.29	0.52*	0.63*
RT_F	0.60*	0.54*	0.63*	0.56*	0.38*	0.28
RT_E	0.55*	0.55*	0.39*	0.28	0.58*	0.69*
LA_DF	0.58*	0.62*	0.48*	0.44*	0.52*	0.69*
LA_PF	0.59*	0.58*	0.69*	0.62*	0.38*	0.34
RA_DF	0.45*	0.48*	0.44*	0.42*	0.30	0.48*
RA_PF	0.54*	0.55*	0.68*	0.56*	0.35	0.37*

*LT_F* left toes flexion, *LT_E* left toes extension, *RT_F* right toes flexion, *RT_E* right toes extension, *LA_DF* left ankle dorsiflexion, *LA_PF* left ankle plantar flexion, *RA_DF* right ankle dorsiflexion, *RA_PF* right ankle plantar flexion.

A detailed examination of Table 3 reveals that the majority of toe and ankle force performance variables exhibited significant correlations with foot length and toe length. For instance, left toe flexion (LT_F) and right toe flexion (RT_F) were both significantly correlated with foot length and toe length, but not consistently with forefoot length.

Regarding the correlations with foot anthropometric measurements, the COP range was positively associated with both foot length and toe length. However, no significant correlation was observed between COPrange and forefoot length. The FFSI did not show any significant association with the foot anthropometric measurements, as presented in Table 4.

**Table 4:** Correlation of anthropometric feet variables with variables of functional balance (*p < 0.05).

Variable	Foot	FFSI	COPrange
Foot length	Left	0.05	0.51*
	Right	0.13	0.56*
Toe length	Left	0.27	0.47*
	Right	0.14	0.46*
Forefoot length	Left	− 0.21	0.35
	Right	− 0.13	0.32

*FFSI* forward functional stability index, *COPrange* displacement range of the center of foot pressure in the LOS test.

As Table 5 indicates, all force variables, except for left ankle dorsiflexion (LA_DF), showed positive correlations with COPrange. Only two force variables, namely left toe flexion force (LT_F) and right toe flexion force (RT_F), demonstrated significant correlations with FFSI.

**Table 5:** Correlation of toe and ankle force performance with variables characterizing functional balance (*p < 0.05).

Variable	COPrange	FFSI
LT_F	0.56*	0.40*
LT_E	0.47*	0.18
RT_F	0.56*	0.38*
RT_E	0.42*	0.13
LA_DF	0.34	0.08
LA_PF	0.50*	0.32
RA_DF	0.41*	0.29
RA_PF	0.45*	0.30

*LT_F* left toes flexion, *LT_E* left toes extension, *RT_F* right toes flexion, *RT_E* right toes extension, *LA_DF* left ankle dorsiflexion, *LA_PF* left ankle plantar flexion, *RA_DF* right ankle dorsiflexion, *RA_PF* right ankle plantar flexion.

## Discussion

This research primarily aimed to evaluate the impact of toe and ankle plantar flexors and dorsiflexors on functional balance. It was hypothesized that a substantial correlation exists between the strength of these flexors and dorsiflexors and functional balance. Additionally, it was theorized that holistic and more focused (isolated) measurements would yield a more comprehensive understanding of the underlying phenomenon.

The preliminary analysis revealed a significant relationship between anthropometric indicators and the strength measures investigated in this research. This result was anticipated because it is based on simple physical principles, i.e., the longer the arm of force, the greater the resultant strength^27^. Conversely, one can argue that an increased support base enhances the system’s stability. However, a broader plane of support (such as a larger foot size) typically correlates with increased body height. This relationship, in turn, often leads to heightened mechanical overloads on the body. The word “overload” here refers to the additional stress or burden placed on the body’s systems. A taller body means more weight and a higher center of mass, which can increase the load on the body’s stabilizing system (specifically, muscles and joints responsible for maintaining balance and posture). Thus, the advantage effect associated with a larger foot is offset by the height of the body—a natural developmental adaptation. In the context of anthropometric measurements, it was found in the current study that the association between these measurements and functional equilibrium parameters was, as anticipated, unrelated to the anthropometric characteristics of the foot. On the other hand, the absolute displacement of COP revealed considerable dependencies. This highlights the favorability of the FFSI, which is designed to precisely ascertain a subject's relative capabilities in terms of functional stability (namely, the assessment of an individual's potential functional forward-leaning capabilities) and is standardized according to the individual's foot length. The method of the FFSI estimation is aimed todetermine the maximum possibilities for each tested individual and the use of these possibilities in percentages by the participants in the LOS test. It is therefore a better measure of functional possibilities than the LOS test alone.

Endo et al.^17^ indicated that the strength of the toe flexor muscles in a healthy foot correlates with the anterio-posterior limit of the functional base of support (FBOS), defined as the maximal volitional anterior and posterior excursion of the ground reaction force, often observed during forward and backward leans. Diverging from previous findings, this work offered a precise description of the FBOS, located at the first metatarsophalangeal joint, which is now referred to as FASL. Such a clarification enables a more precise evaluation of an individual's functional ability. In this analysis, young and healthy participants uniformly reached their individual FASL, in line with the expected outcomes for this demographic^7^. Endo et al.’s^17^ investigation, echoing the findings of the current study, demonstrated that the greater the strength of the toe flexor muscles, the more the ground reaction force that can be moved forward under the foot. The presented approach offers an enhanced a technique for precisely measuring the force exerted by toe and ankle muscles during flexion and extension. This was achieved by isolating specific muscle activities, deliberately excluding any synergistic influences that could distort the results. This approach offers a direct evaluation, in contrast to the indirect approach employed by Endo et al.^17^. The method was developed to measure the exerted force of toe and ankle muscles in flexion and extension directly, enabling the isolation of specific muscle activities and exclusion of synergistic muscles that might influence the outcome. This approach provides a more immediate assessment than the indirect method used by Endo et al.^17^. This progression aimed to enhance the trustworthiness of the measurements. Notably, Endo et al. reported no reliability measurements, underscoring the potential benefit of the current study’s direct measurement approach in contributing to a more robust understanding of muscle force dynamics. Similarly to previous studies concerning FFSI, the current study reported high reliability of the measurements concerning muscle force production^7^. Interestingly, a lack of significant correlations was observed between functional stability, as expressed by the index, and ankle muscle strength. Such a correlation would seem intuitive; however, statistically significant correlations were evident only in the case of toe muscles. This is certainly proof that more isolated diagnostics are of significance.

A positive correlation was observed between foot length, toe length, and functional balance features, but not between FFSI and foot measurements. Two relationships were found between force performance (left and right toe flexion forces) and FFSI. Despite the correlations being mostly moderate, the relationship between toe flexor strength and FFSI holds functional significance, particularly in specific contexts or populations that may be examined in future studies. The information in this manuscript carries an important and utilitarian message, previously presented in the literature, albeit approached differently.

## Conclusions

The results demonstrated a substantial link between anthropometric indicators and the investigated strength measures, as well as a favorable correlation between foot and toe length and functional balancing aspects. However, no association was detected between FFSI-measured functional balance and anthropometric foot measurements. Correlations between FFSI and strength were noted only in toe flexors. In addition, the study demonstrated the applicability of the FASL, located at the first metatarsophalangeal joint, which enabled an accurate calculation of functional balancing capacity. Overall, the work offered substantial insights into the relationship between muscle strength and functional balance, suggesting areas for further research and clinical evaluation. The logical next step would be to examine specific age groups to confirm these results and introduce some targeted interventions aimed at balance enhancement.

### Limitations of the study

Despite the study’s contributions, there were limitations: the lack of diversity and the limited assessment of balance, which did not encompass flexibility. The study focused on one measure of functional balance (FFSI), which may not fully capture all aspects of balance. Additional measures may provide a more comprehensive understanding of the relationship between muscle strength and functional balance. Future research ought to focus on an older adult population, enabling a clearer understanding of the relationship between muscle strength and the risk of falls.

## Data Availability

The datasets used and/or analyzed during the current study are available from the corresponding author upon reasonable request.
